# Regenerative Potential of Mesenchymal Stromal Cells: Age-Related Changes

**DOI:** 10.1155/2016/1461648

**Published:** 2016-05-09

**Authors:** Flavia Bruna, David Contador, Paulette Conget, Benjamín Erranz, Claudia L. Sossa, Martha L. Arango-Rodríguez

**Affiliations:** ^1^Center for Regenerative Medicine, School of Medicine, Clínica Alemana, Universidad del Desarrollo, Lo Barnechea, 7710162 Santiago, Chile; ^2^Production Unity of Advanced Therapy, Fundación Oftalmológica de Santander, Clínica Carlos Ardila Lulle (Foscal Internacional), Universidad Autónoma de Bucaramanga (UNAB), 681004153 Bucaramanga, Colombia

## Abstract

Preclinical and clinical studies have shown that a therapeutic effect results from mesenchymal stromal cells (MSCs) transplant. No systematic information is currently available regarding whether donor age modifies MSC regenerative potential on cutaneous wound healing. Here, we evaluate whether donor age influences this potential. Two different doses of bone marrow MSCs (BM-MSCs) from young, adult, or old mouse donors or two doses of their acellular derivatives mesenchymal stromal cells (acd-MSCs) were intradermally injected around wounds in the midline of C57BL/6 mice. Every two days, wound healing was macroscopically assessed (wound closure) and microscopically assessed (reepithelialization, dermal-epidermal junction, skin appendage regeneration, granulation tissue, leukocyte infiltration, and density dermal collagen fibers) after 12 days from MSC transplant. Significant differences in the wound closure kinetic, quality, and healing of skin regenerated were observed in lesions which received BM-MSCs from different ages or their acd-MSCs compared to lesions which received vehicle. Nevertheless, our data shows that adult's BM-MSCs or their acd-MSCs were the most efficient for recovery of most parameters analyzed. Our data suggest that MSC efficacy was negatively affected by donor age, where the treatment with adult's BM-MSCs or their acd-MSCs in cutaneous wound promotes a better tissue repair/regeneration. This is due to their paracrine factors secretion.

## 1. Introduction

Many preclinical studies have demonstrated that mesenchymal stromal cells (MSCs) enhance a regenerative wound microenvironment. In different animal models, such as full thickness excisional wound [1], diabetic foot ulcer [2], pressure ulcers [3], and burn injury [4], the administration of MSCs improves wound healing, by means of the increased accelerated wound closure, reduced scarring, promoting of collagen synthesis, angiogenesis, and improved tensile strength.

On the other hand, several clinical trials have shown that MSCs are safe and therapeutic in the treatment of chronic wounds [5], limb ischemia [6], diabetic foot ulcer [7], and radiation burn [8]. These studies reported that administration of MSCs has produced clinical improvements including increased perfusion, decreased pain, reduction of ulcer size, and modulating of the radiation inflammatory processes and an improvement of wound repair, respectively.

Although the mechanisms by which MSCs ameliorate tissue damage have been the subjects of debate for years, two theories currently offer explanations for these cells' therapeutic effects: (i) their differentiation or transdifferentiation into parenchymal cells [9] or (ii) their bioactive soluble factor production (growth factors, cytokines, and specific proteins) [10].

Historically, MSCs were considered to be hypoimmunogeneic because MSCs exhibit low levels of expression of MHC-I, no expression of either MHC class II markers, and costimulatory molecules, which allows them to avoid immunosurveillance [11]. Therefore, allogeneic MSCs could be more effective than the use of patient-derived dysfunctional autologous MSCs. In this context, an aspect of relevant importance donor age-related differences. Even though it has long been known that advanced age is negatively correlated with an organism's reparative and regenerative potential, scarce, conflicting systematic information is available concerning the effects of donor age on MSC regenerative potential. Several* in vitro* reports involving various species (rodents, monkeys, and humans) have indicated that aging is accompanied by many changes in biological processes affecting MSCs such as (i) a decline in the number of cells obtained by bone marrow aspiration, (ii) decrease in their proliferation and differentiation potential, (iii) decreased CFU frequency, (iv) increased senescent cell frequency, (v) lack of the characteristic spindle-like morphology observed in younger donors' MSCs, (vi) decrease in the expression of MSC surface markers, and (vii) miRNA expression and immunomodulatory potential [12–14].

On the other hand, few recent* in vivo* studies have demonstrated that the therapeutic effectiveness of MSCs is dependent on the age of the donor. In acute myocardial infarction model rats, a more rapidly decreased survival rate of the old MSCs in the infarcted region was observed, which may be caused by the increased susceptibility of the cells to reactive oxygen species [15]. Another study into chronic experimental autoimmune encephalomyelitis mouse model demonstrated that donor age significantly affects the ability of human MSCs to provide neuroprotection, immunomodulation, and/or remyelination [16]. Also, it has been reported that the aging of human MSCs impairs angiogenic capacities in a mouse ischemic hindlimb model, since it has effects on their ability to differentiate towards endothelial cells, secretion of proangiogenic and prosurvival factors, and oxidative stress [17]. However, insufficient information is available concerning the effects of donor age on MSC regenerative potential in cutaneous wound healing [14]. In the present study, we evaluate whether donor age influences MSC regenerative potential in cutaneous wound healing in a full thickness excisional wound model [18]. Every two days after MSC transplant, wound healing was macroscopically (wound closure) and microscopically assessed (reepithelialization, dermal-epidermal junction, skin appendage regeneration, granulation tissue formation, leukocyte infiltration, and density dermal collagen fibers) at 12 days after MSC transplant. The results suggest that the treatment with adult's BM-MSCs or their acd-MSCs in cutaneous wound promotes tissue repair/regeneration. The mechanisms involved in these beneficial effects were likely related to the ability of adult BM-MSCs to release paracrine factors in a regulated way, thereby modulating the wound healing response.

## 2. Materials and Methods

### 2.1. Animals

C57BL/6 mice (Jackson Laboratory, Bar Harbor, ME) were used in our experiments; they were kept at constant temperature and humidity, using a 12 : 12-hour light-dark cycle, and had unrestricted access to a standard diet and water. The animals were anesthetized with 1.1 mg/Kg 2,2,2-tribromoethanol (Avertin, Sigma-Aldrich) or sevoflurane (Abbott, Japan) when required; all animal procedures were approved by the Ethic Committee of Facultad de Medicina, Clínica Alemana, Universidad del Desarrollo (approval ID:2011-14).

### 2.2. Excisional Skin Wound Model

Eight-week-old female C57BL/6 mice (19–22 g body weight) were anesthetized with Avertin and the hair was removed from the dorsal surface; 6 mm full thickness punch biopsy wounds were made on one side of the midline extending through the panniculus carnosus as has been described by Galiano et al. [18].

The number of animals used in BM-MSCs experimental groups was vehicle (*n* = 15), 0.5 × 10^6^ BM-MSCs isolated from young (*n* = 6), adult (*n* = 6), and old (*n* = 6), and 1 × 10^6^ BM-MSCs isolated from young (*n* = 7), adult (*n* = 6), and old (*n* = 6).

The number of animals used in acd-MSCs experimental groups was vehicle (*n* = 12), 1x dose of acd-MSCs from BM-MSCs from young (*n* = 6), adult (*n* = 6), and old donors (*n* = 6), and 6x dose of acd-MSCs from BM-MSCs from young (*n* = 6), adult (*n* = 6), and old donors (*n* = 6).

### 2.3. MSC Administration

Around the wound at four injection sites, vehicle (control-saline solution with 5% autologous plasma) or 0.5 × 10^6^ or 1 × 10^6^ BM-MSCs derived from young (8 weeks), adult (33 weeks), or old donors (50 weeks) male C57BL/6 mice in 60 *μ*L vehicle or two doses (1x or 6x) of 100 *μ*L of acd-MSCs were administered intradermally. Tegaderm (3M, London, ON, Canada) was immediately placed over the wounds; the animals were housed individually.

### 2.4. Wound Healing Follow-Up

The wound area was measured in duplicate with a digital calliper (Mitutoyo Sul Americana LTDA, Brazil) every two days after BM-MSCs from different ages or their acd-MSCs transplant and digital photographs were taken (FUJIFILM-Finepix HS20 EXR). The wound area was analyzed by the measure obtained with the digital caliper. Time elapsed to wound closure was defined as the time during which the wound bed became completely reepithelialized and filled with new tissue. The percentage of wound closure was calculated as follows: (area of original wound − area of actual wound)/area of original wound × 100. All wounds were repaired after 12 days and the mice were sacrificed; skin samples from the affected area and 2 mm of the surrounding skin were harvested using an 8 mm biopsy punch.

### 2.5. Histological Assessment

Mice were sacrificed by an overdose of anesthesia (ketamine 50 mg/kg-xylazine 5 mg/kg (Centrovet)). The skin was rapidly removed, fixed in 10% formalin, and embedded in paraffin. Four *μ*m thick skin sections were stained with haematoxylin-eosin (Sigma-Aldrich, St. Louis, MO) and Masson's trichrome (Diagnostic Biosystems, Pleasanton, CA). Computational analysis was made to provide quantitative analysis regarding the dermoepidermal junction. In short, the analysis was made by manually tracing the border of area with incomplete junction and calculating pixel area using an image analysis program (NIH ImageJ). The size of the traced area was calculated automatically by the software. The graph represents the frequency (%) of animals with different scores. We gave the following scores.

0: >2,801 pixels per area, corresponding to injunction; I: 1,201–2,800 pixels per area; II: 751–1,200 pixels per area; III: 401–750 pixels per area; and IV: 0.1–400 pixels per area, corresponding to complete junction (Supplementary Figure  1A in Supplementary Material available online at http://dx.doi.org/10.1155/2016/1461648).

We standardized histopathologic criteria in haematoxylin-eosin for determining the degree of leukocyte infiltration from − to +++ (unquantifiable subjective parameters) (Supplementary Figure  1B).

Also, a quantification of granulation tissue was made by manually tracing the border of area of granulation tissue and calculating pixel area using an image analysis program (NIH ImageJ). The size of the traced area was calculated automatically by the software. Also, we made a classification of granulation tissue type.

Appendage-like structures in each wound section were observed with Masson's trichrome staining.

Morphometric computational analysis of dermal collagen was made in Masson's trichrome staining to provide quantitative analysis regarding the density and the intensity of dermal collagen fibers according to method described by Miot and Brianezi [19].

Tissue sections of 4 *μ*m were evaluated by epidermal keratin subunits. The histological samples were permeabilized with TBS-Tween 0.1% (Sigma-Aldrich, St. Louis, MO) and afterwards were blocked with 5% fetal bovine serum (Gibco, Auckland, NZ). Keratinocytes were stained with a monoclonal antibody against epidermal keratin subunits (Abcam Inc., Cambridge, MA) and visualized with a secondary antibody: Alexa Fluor 647 (Abcam Inc., Cambridge, MA, USA). Cross-reactivity of the secondary antibody was tested without primary antibody. DAPI (4′6-diamidino-2-phenylindole) (AppliChem, Germany) was used for nuclear staining. All slides were examined under a Leica DM2000 microscope (Wetzlar, Germany) and images captured with a Leica DFC 295 camera (Wetzlar, Germany).

### 2.6. Isolation and* ex vivo* Expansion of Mouse BM-MSCs

Bone marrow cells were collected by flushing the femurs and tibias from C57BL/6 male mice of different ages: young, adult, and old. Bone marrow cells were obtained with minimal essential medium (Alpha-MEM; Gibco, Auckland, NZ). The cells were cultured in Alpha-MEM supplemented with 10% fetal bovine serum (HyClone Laboratories, Logan, Utah) and 8 *μ*g/mL gentamicin (Sanderson Laboratory, Chile) after centrifugation and plated at 1 × 10^6^ nucleated cells/cm^2^ density. Nonadherent cells were removed 72 hours later and fresh medium was added to the cells; adherent cells were detached after 96 hours with 0.25% trypsin, 2.65 mM EDTA (Life Technologies, Grand Island, NY) spun and subcultured at 7,000 cells/cm^2^. Adherent cells were characterized and transplanted after one subculture.

### 2.7. Obtension of acd-MSCs

The acd-MSCs were produced as follows: BM-MSC culture passage 1, reaching 80% confluence, in 10 cm tissue culture dishes, was fed with 3 mL per dish of serum-free Alpha-MEM and incubated for 24 hours. The medium was collected and spun to remove all cell debris; this medium was called acd-MSCs and further concentrated (6-fold) by filtration using centrifugal filter units with 3 KDa cut-off (Millipore) following the manufacturer's instructions.

### 2.8. MSC Characterization

#### 2.8.1. CFU Assay

Bone marrow cells were suspended in Alpha-MEM and supplemented and plated at 17,000 nucleated cells/cm^2^ density in triplicate. Nonadherent cells were removed with fresh medium, which was changed twice a week. Cells were stained with 0.5% crystal violet (Sigma-Aldrich, St. Louis, MO) in 10% methanol for 20 minutes on day 21. The colonies formed were counted after four washes with phosphate buffered saline (Life Technologies Grand Island, NY) and the results were expressed as CFU/million nucleated cells plated.

#### 2.8.2. Proliferation Assay

First passaged BM-MSCs were subcultured at 4,000 cells/cm^2^; the medium was changed every three days. The amount of cells was determined on days 0, 3, 6, 9, and 12 after staining with 0.5% crystal violet in 10% methanol for 20 minutes. Cell-incorporated crystal violet was solubilized after four washes by incubation with phosphate buffer in methanol (50 : 50) and spectrophotometrically quantified (570 nm absorbance).

#### 2.8.3. MSC Differentiation Assay

BM-MSCs were cultured either in osteogenic (0.1 *μ*M dexamethasone, 10 mM *β*-glycerophosphate, 50 *μ*g/mL ascorbic acid [20]) or in adipogenic (1 *μ*M dexamethasone, 100 *μ*g/mL 3-isobutyl-1-methylxanthine (IBMX) [21]) medium for 21 days, respectively.

#### 2.8.4. Immunophenotyping

Although there is no current consensus with respect to murine MSCs markers (versus those for human MSCs), immunophenotyping was performed by flow cytometry analysis. Cells were incubated with anti-CD45.2, clone 104 (APC-eFluor780 conjugated) (eBioscience, San Diego, CA), anti-CD11b, clone M1/70 (PE-Cy5-conjugated) (eBioscience), anti-Sca-1, clone D7 (PE-conjugated) (eBioscience), anti-CD90.2, clone 53-2.1 (PE-Cy7-conjugated) (BD Pharmingen*™*), and anti-ASMA, clone 1A4 (FITC-conjugated) (Sigma).

Statistical data of flow cytometry were showed in Supplementary Table  1.

#### 2.8.5. Gene Expression Analysis

Total RNA was isolated from 1 × 10^6^ mouse BM-MSCs isolated from different aged donors using trizol reagent (Invitrogen, Carlsbad, CA). RNA concentration was determined at 260 nm absorbance using a Nanodrop 2000 spectrophotometer (Thermo Scientific, USA). One *μ*g of total RNA was treated with grade I DNase (Invitrogen, Carlsbad, CA) and reverse transcribed with 200 U M-MLV reverse transcriptase (Promega, Madison, WI) using 300 pmol oligo-dT. A LightCycler thermocycler (Roche, Indianapolis, IN) was used for RT-PCR with 50 *η*g cDNA in 10 *μ*L final volume of PCR LightCycler-DNA Master SYBRGreen reaction mix (Roche, Indianapolis, IN), 2.5 mM MgCl_2_, and 0.5 *μ*M of each specific primer (Supplementary Table  2). Controls without reverse transcription were included to ensure that amplification was from mRNA and not from genomic DNA. Amplicons were characterized according to their melting temperature, determined in the LightCycler thermocycler; their size was evaluated by electrophoresis on agarose gel. Each target gene's mRNA level was standardized against reference gene GAPDH for each sample. The ΔCt method was used for quantification and values were expressed as relative fold change [22].

#### 2.8.6. ELISA

The acd-MSCs samples from donors of different ages were subjected to ELISA for their content of specific growth factors and proteins relevant to wound healing. Factors such as VEGF, ANG-2, IGF-1, KGF, HGF, CoL-1, MMP-1, and PGE_2_ (ELISA kits from R&D Systems, Abcam, and MyBioSource) were measured following the manufacturer's instructions.

### 2.9. Statistical Analysis

All animal data values were expressed as the mean ± SEM. ANOVA was used for multiple group comparison, followed by Dunn's multiple comparison test. A <0.05 probability *p* value was considered statistically significant.

## 3. Results

### 3.1. Adult Donor Syngeneic BM-MSCs Accelerated Wound Closure Kinetics

In order to study the activity of BM-MSCs from different age donors on wound closure, two different doses of syngeneic BM-MSCs derived from different aged donor mice were injected into excisional wounds in C57BL/6 mice. There was accelerated wound closure with syngeneic BM-MSCs-treated wounds compared to vehicle-treated wounds (8 versus 12 days, resp.). Moreover, BM-MSCs-induced enhancement appeared early after 2 days and became more evident 4 days after cell injection, compared to vehicle. Furthermore, it was found that BM-MSCs obtained from adult donors were the most efficient tools for fastening wound closure (50% after 4 days for adult versus 6 for young and old); such difference was not improved by increasing the amount of cells.

**Figure 1:**
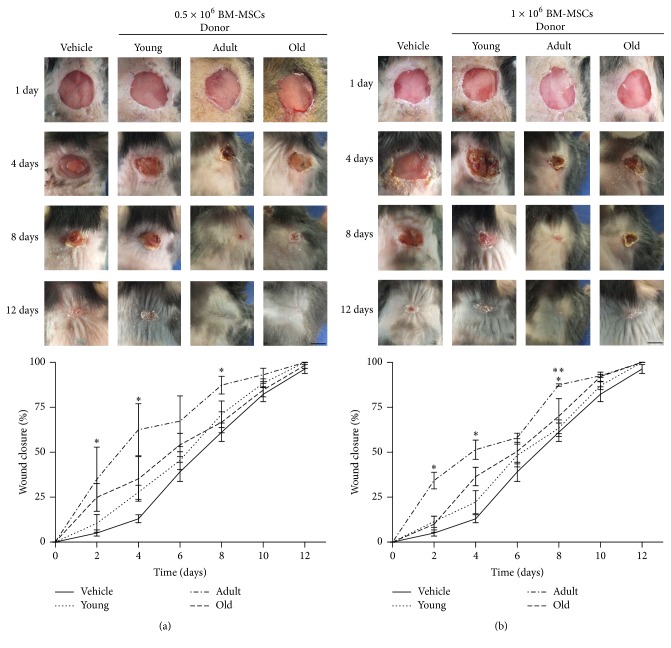
Adult donor syngeneic BM-MSCs accelerated wound closure kinetics. A murine excisional wound model in which wounds were treated with (a) 0.5 × 10^6^ and (b) 1 × 10^6^ BM-MSCs isolated from female young, adult, and old C57BL/6 or 5% autologous plasma in physiological saline (vehicle). Representative photographs of wounds in C57BL/6 before treatment or 4, 8, and 12 days afterwards. Wound size measurement at different times. Data are mean ± SEM (vehicle (*n* = 15); 0.5 × 10^6^ BM-MSCs isolated from young (*n* = 6), adult (*n* = 6), and old (*n* = 6) and 1 × 10^6^ BM-MSCs isolated from young (*n* = 7), adult (*n* = 6), and old (*n* = 6)). ANOVA^*∗*^  (*p* < 0.05): young, adult, and old versus vehicle. ANOVA^*∗∗*^  (*p* < 0.001): adult versus young and old.

Significant differences between different age groups regarding wound closure were only found after day 8 with high doses of syngeneic BM-MSCs (adult versus young and old) (Figures 1(a) and 1(b)).

### 3.2. Syngeneic BM-MSCs Led to the Recuperation of Quality Regenerated Skin

**Figure 2:**
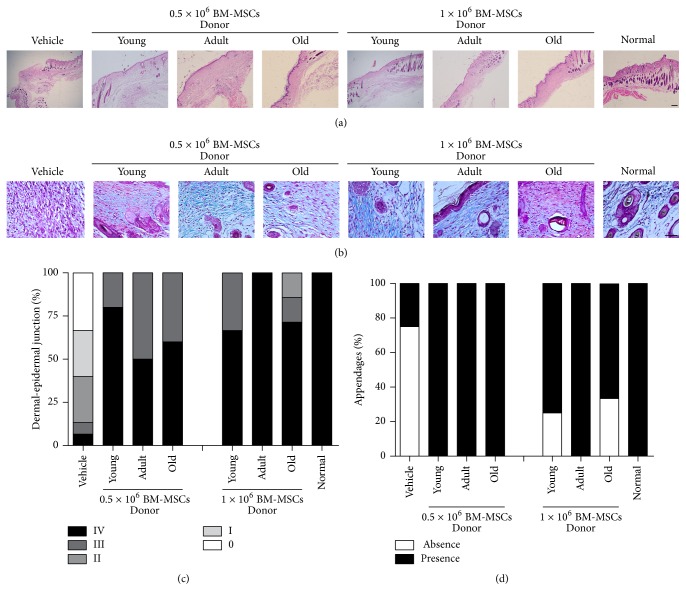
Syngeneic BM-MSCs led to the recuperation of quality regenerated skin. Histological section images 12 days after treatment. (a) Haematoxylin-eosin stain that represents the dermoepidermal junction. Scale bar 100 *μ*m. (b) Masson's trichrome stain that shows appendage-like structure in the dermis. Scale bar 50 *μ*m. Frequency (%) of animals with (c) different histological scores of dermoepidermal junction and (d) different histological scores of appendage structure. Representative results for 6–15 animals per experimental group.

In order to determinate the quality of regenerated skin with BM-MSCs from different age donors, we evaluated reepithelialization, reconstitution of the dermoepidermal junction, and new skin appendage structure on day 12 in cutaneous lesion. A difference in wound reepithelialization was observed in lesions, which received BM-MSCs compared to lesions which received only the vehicle (Figure 2(a)). Moreover, all the normal layers of the epithelium could already be observed in the wounds treated with any of different aged BM-MSCs on day 12 compared to wounds treated with vehicle (Figure 2(a)). Greater reconstitution of the dermoepidermal junction was observed in mice treated with both doses of BM-MSCs (100% score III to IV) compared to animals treated with vehicle (13% score III to IV) (Figure 2(c)).

Other signs of skin regeneration, such as new skin appendage structure (hair follicles or sebaceous glands), were observed in the wounds treated with BM-MSCs obtained from donors of different ages compared to wounds treated with just vehicle (Figures 2(b) and 2(d)).

### 3.3. Syngeneic BM-MSCs Led to Skin Healing Process

To evaluate whether BM-MSCs from different ages could induce the recovery of healing process, we determined the amount of granulation tissue formation and type, inflammatory infiltrates, and density of the collagen fibers in cutaneous lesion. A difference in the amount of granulation tissue formation was observed on day 12 concerning cutaneous lesions which received BM-MSCs (mean = 69030–163375 area of pixels) compared to lesions that received only the vehicle (mean = 56746). Greater granulation tissue formation in the BM-MSCs-treated groups was observed in mice treated with doses 0.5 × 10^6^ young and adult or 1 × 10^6^ old BM-MSCs (mean = 136213–163375 area of pixels).

**Figure 3:**
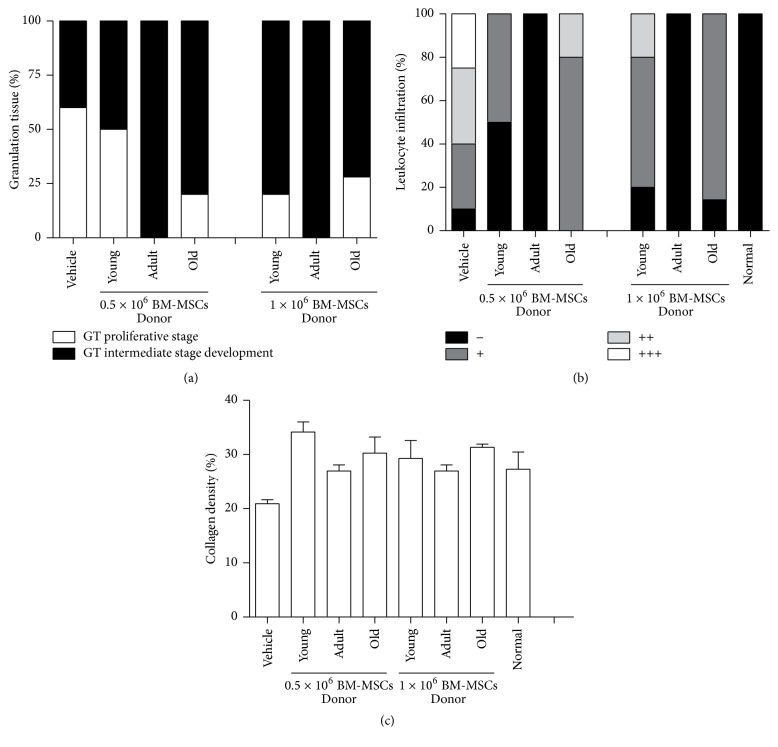
Syngeneic BM-MSCs led to skin healing process. Frequency (%) of animals with different histological scores of (a) granulation tissue formation type and (b) leukocyte infiltration in the dermis. (c) Quantitative analysis of density of dermal collagen fibers. Representative results for 6–15 animals per experimental group.

More granulation tissue in intermediate stage development with less number of blood vessels, discrete fibroblast proliferation, and collagen deposition mature type were found in the dermal connective tissue of all animals treated with adult BM-MSCs on day 12 (100%), compared to group treated with young and old BM-MSCs (63–75%) or vehicle (40%) where the granulation tissue was in the proliferative phase with abundant fibroblastic proliferation type and greater number of blood vessels and inflammatory process with acute activity (Figure 3(a)).

More inflammatory infiltrates were found in the dermal connective tissue of vehicle-treated groups on day 12 (90% from score + to +++), young BM-MSCs-treated groups with both doses of cells (50% and 80% score + to ++), and old BM-MSCs-treated groups with both doses (85 and 100% score + to ++) compared to adult BM-MSCs-treated groups with both doses where cell infiltration was reduced 100% to score (−) (Figure 3(b)).

Greater density of the collagen fibers was found in the BM-MSCs-treated groups (mean = 26–34%), suggesting more intense remodeling 12 days after the lesion compared to vehicle group (mean = 20%) having less remodeling, as indicated by thinner and less densely organized fibers (Figure 3(c)).

### 3.4. Characterizing BM-MSCs Isolated from Different Aged Mice

**Figure 4:**
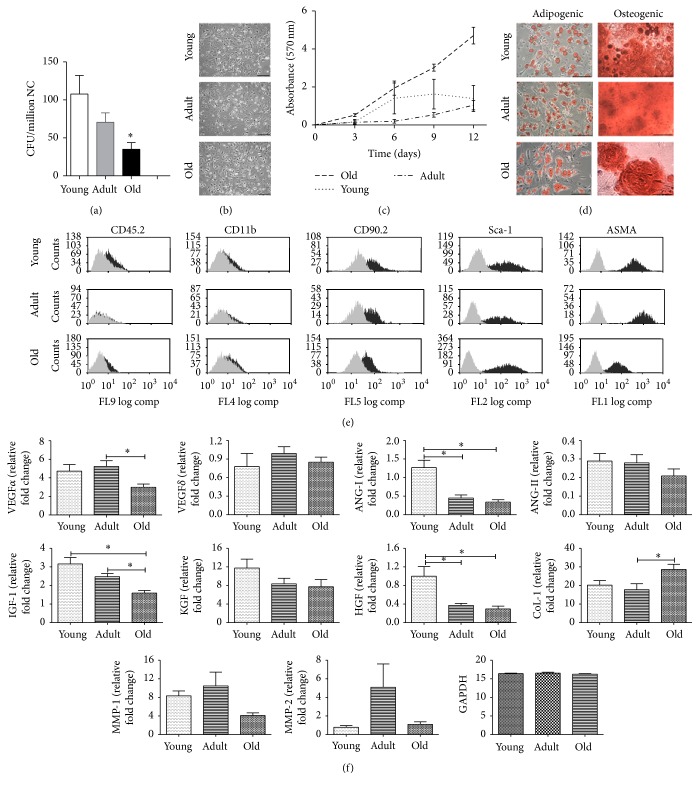
Characterization of isolated BM-MSCs from different aged C57BL/6 mice. (a) Bone marrow abundance was determined by CFU assay. Data are mean ± SEM (*n* = 6). ANOVA^*∗*^: young versus old. (b) Morphology from MSC derived from different age groups. Scale bar 100 *μ*m. (c) Proliferation kinetics was evaluated by crystal violet staining (570 nm absorbance) over a period of 12 days. Data are mean ± SEM (*n* = 4). (d) Differentiation potential was assessed by* in vitro* exposure to adipogenic or osteogenic medium and stained with Oil Red and Alizarin Red after 21 days, respectively. (e) Flow cytometry was used for the assessment of MSC surface markers. MSCs expressed anti-CD90.2, anti-Sca-1, and anti-ASMA antibodies but not anti-CD45.2 and anti-CD11b antibodies. Representative images for 2 animals per each age group. Representative images from 3 animals per each age group. Scale bar 100 *μ*m. (f) The mRNA profiles of MSC paracrine factors (VEGF*α*, VEGF*δ*, ANG-I, ANG-II, IGF-1, KGF, HGF, CoL-1, MMP-1, and MMP-2) from different aged donors were analyzed by RT-PCR and normalized by their relative ratio to GAPDH. Data are mean ± SEM (*n* = 8). GAPDH, glyceral-dehyde-3-phosphate dehydrogenase. ^*∗*^ *p* < 0.05.

To approach whether the changes observed on wound closure kinetics, quality regenerated skin, and healing process from BM-MSCs of different age could be by* in vitro* culture on the basic properties of them, we characterized and compared cells isolated from young, adult, or old age donors. Viable BM-MSCs were significantly more abundant in the bone marrow of young donors than adult or old ones (109 CFU/million nucleated cells versus 72 and 34, resp.) (Figure 4(a)).

Any change in the morphology of the cells at day 12 of culture was observed and the size of the BM-MSCs in the three age groups was similar (31–36 *μ*m) (Figure 4(b)).

BM-MSCs obtained from three ages donors had an initial growth phase similar in culture (3 days); however, old BM-MSCs growth kinetics were higher than those for young and adult BM-MSCs, although such difference was not statistically significant (Figure 4(c)). Additionally, BM-MSCs isolated from three different age donors were able to differentiate into adipocytes and osteocytes (Figure 4(d)).

BM-MSCs isolated from three ages on early passage (one passage cells) were found to be positive for anti-CD90, anti-Sca-1, and anti-ASMA antibodies but not for anti-CD45 and anti-CD11b antibodies. No significant age-related changes were found in the marker expression levels between the three age groups (Figure 4(e) and Supplementary Table  1).

Gene expression profile from BM-MSCs of different age donors was determined for following growth factors and specific proteins' relative mRNA expression levels: VEGF*α*, VEGF*δ*, ANG-I, ANG-II, IGF-1, KGF, HGF, CoL-1, MMP-1, and MMP-2.  Figure 4(f) shows that KGF, VEGF*δ*, ANG-II, MMP-1, and MMP-2 mRNA expression levels were not significantly different from those for BM-MSCs from donors of all three ages, whereas IGF-1 (2–1.5-fold) and VEGF*α* (1.75-fold) expression levels were higher in BM-MSCs from young and adult donors compared to ANG-I (2.86-fold) and HGF (2.7-fold), where expression levels were only significantly higher in young donors. On the other hand, the highest CoL-1 expression level (1.61-fold) was observed in BM-MSCs from old donors. EGF expression level was not observed in BM-MSCs from different aged donors.

### 3.5. Adult Donor acd-MSCs Accelerated Wound Closure Kinetics

**Figure 5:**
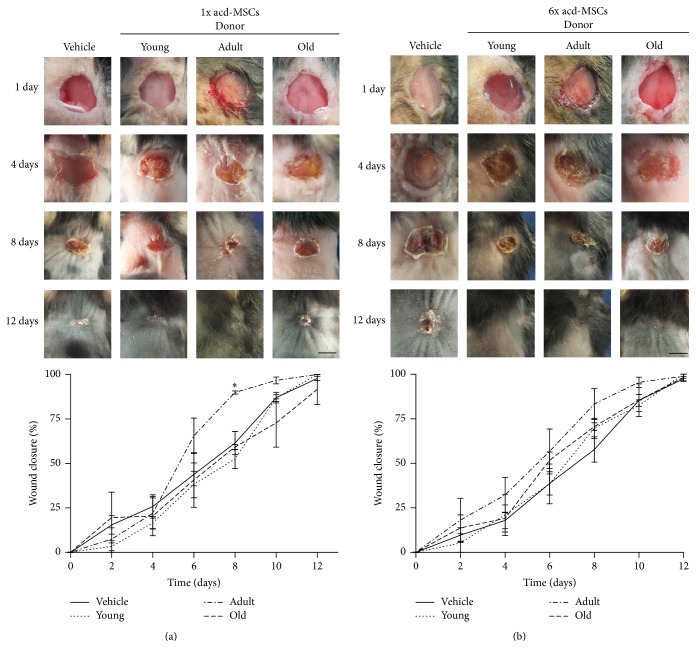
Adult donor acd-MSCs accelerated wound closure kinetics. Murine excisional wound model in which wounds were treated with (a) 1x dose and (b) 6x dose of acd-MSCs from young, adult, and old donors' BM-MSCs or Alpha-MEM medium (vehicle). Representative photographs of C57BL/6 wounds before treatment or 4, 8, and 12 days afterwards. Measurement of wound size at different times. Data are mean ± SEM (vehicle *n* = 12; 1x dose of acd-MSCs from BM-MSCs from young (*n* = 6), adult (*n* = 6), and old donors (*n* = 6) and 6x dose of acd-MSCs from MSCs from young (*n* = 6), adult (*n* = 6), and old donors (*n* = 6)). ANOVA^*∗*^  (*p* < 0.0): young, adult, and old versus vehicle.

In order to determinate whether the paracrine effect of BM-MSCs was affected by age donors, we evaluate the activity of acd-MSCs from different age donors on wound closure. There was accelerated wound closure in C57BL/6 mice wounds treated with 1x dose of adult donor acd-MSCs compared to lesions which only received the vehicle or young and old donors' acd-MSCs (8 versus 12 days, resp.). The enhancement appeared 6 days after treatment in C57BL/6 mice and became more evident on day 8. It was found that acd-MSCs from young and old donors MSCs were the least efficient condition for fastening wound closure (50% on day 6 for young and old versus day 5 for adult donors). This difference was improved by increasing old donor doses (50% on day 6 for old donors versus day 7 for young donors) (Figures 5(a) and 5(b)).

### 3.6. acd-MSC Led to the Recuperation of Quality Regenerated Skin

**Figure 6:**
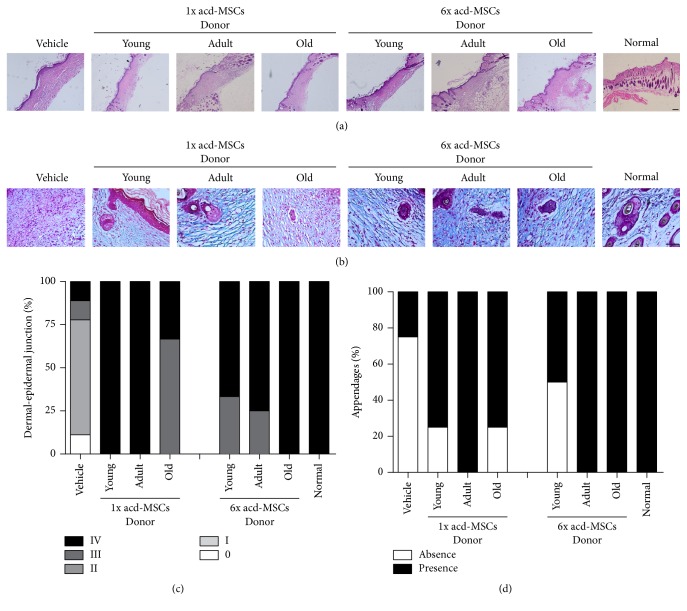
acd-MSCs led to the recovery of quality regenerated skin. Histological section images 12 days after treatment. (a) Haematoxylin-eosin stain that represents the dermoepidermal junction. Scale bar 100 *μ*m. (b) Masson's trichrome stain that showed appendage-like structure in the dermis. Scale bar 50 *μ*m. Frequency (%) of animals with (c) different histological scores of dermoepidermal junction and (d) histological scores of appendage structure. Representative results for 6–12 animals per experimental group.

In order to determinate the quality of regenerated skin with acd-MSCs from different age donors, we evaluate reepithelialization, reconstitution of the dermoepidermal junction, and new skin appendage structure on day 12 in cutaneous lesion. A difference in wound reepithelialization was observed between cutaneous lesions which had received acd-MSCs compared to lesions which had only received the vehicle (Figure 6(a)). Greater reconstituted dermoepidermal junction (100% score III to IV) was observed in all mice treated with acd-MSCs (both doses) than in animals treated with vehicle had smaller percentage (22% score III to IV) (Figure 6(c)).

acd-MSCs obtained from donors of different ages contributed towards appendage-like structure formation in the regenerated dermis compared to wounds treated with just vehicle (Figures 6(b)–6(d)).

Pan-cytokeratin (an epithelial tissue marker) expression was found in all groups (BM-MSCs, acd-MSCs, and vehicle) (Supplementary Figure  S2).

### 3.7. acd-MSCs Led to Skin Healing Process

To evaluate whether acd-MSCs from young, adult, or old age could induce the recovery of healing process, we determined the amount of granulation tissue formation, inflammatory infiltrates, and density of the collagen fibers in cutaneous lesion. A difference in the amount of granulation tissue formation was observed on day 12 concerning cutaneous lesions, which received acd-MSCs compared to lesions which received only the vehicle, although such difference was not statistically significant. Greater granulation tissue formation was observed in animals treated with 1x doses of young, adult, and old and 6x doses of adult and old acd-MSCs (mean = 89553–123583 area of pixels) compared to animals treated with vehicle or 6x doses of young acd-MSCs (mean = 65459–66309 area of pixels).

**Figure 7:**
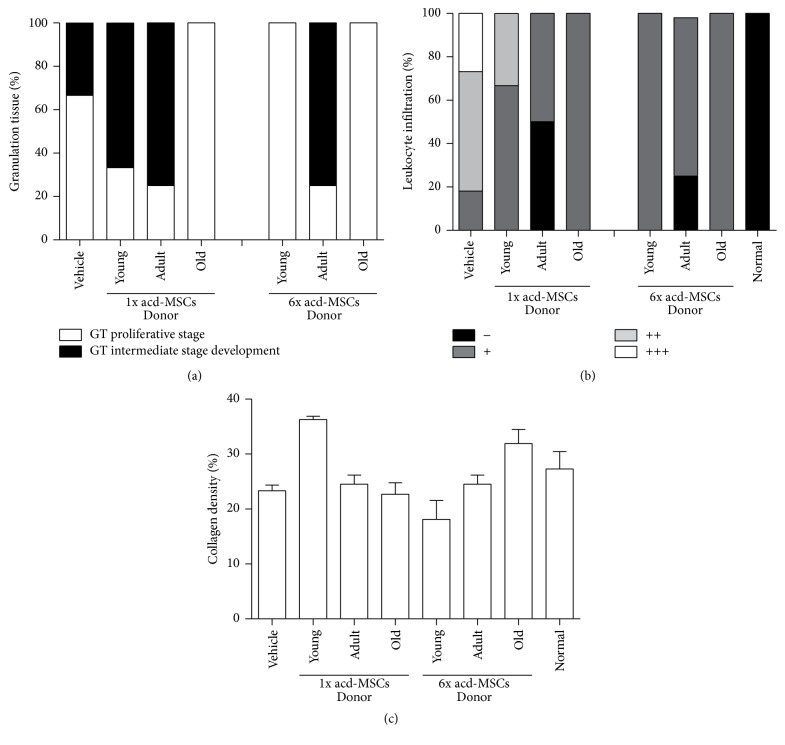
acd-MSCs led to skin healing process. Frequency (%) of animals with different histological scores of (a) granulation tissue formation type, (b) leukocyte infiltration in the dermis, and (c) quantitative analysis of density of dermal collagen fibers. Representative results for 6–12 animals per experimental group.

More granulation tissue formation in intermediate stage development with less number of blood vessels, discrete fibroblast proliferation, and collagen deposition mature type were found in the dermal connective tissue of all animals treated with adult acd-MSCs on day 12 (75%), compared to group treated with young (0–66%) and old acd-MSCs (0%) or vehicle (33%), where the granulation tissue was proliferative phase with abundant proliferation fibroblastic type and greater number of blood vessels and inflammatory process with acute activity (Figure 7(a)).

More leucocyte infiltration was found in the dermal connective tissue of vehicle-treated groups on day 12 (82% from score ++ to +++), young acd-MSCs-treated groups with both doses (100% from score + to ++), and old acd-MSCs-treated groups with both doses (100% from score +) compared to groups treated with adult acd-MSCs with both doses where cell infiltration was reduced 25–50% score (–) (Figure 7(b)).

Greater density of the collagen fibers was found in the young and old acd-MSCs-treated groups (mean = 31–36%) and was dose dependent, suggesting more intense remodeling 12 days after the lesion compared to adult acd-MSCs or vehicle group (mean = 18–23%) having less remodeling and less densely organized fibers (Figure 7(c)).

### 3.8. acd-MSCs Secretion Profile

**Figure 8:**
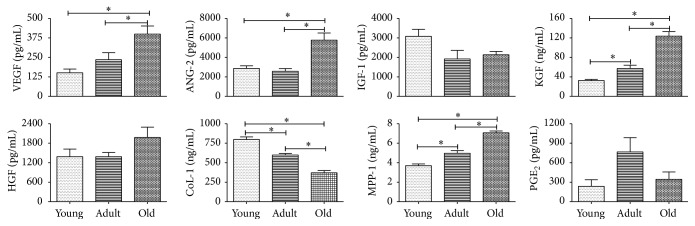
acd-MSCs secretion profile. The secretion levels of MSC paracrine factors (VEGF, ANG-2, IGF-1, KGF, HGF, CoL-1, MMP-1, and PGE_2_) from different aged donors were analyzed by ELISA. Data are mean ± SEM (*n* = 8). ANOVA^*∗*^: young versus adult versus old. Only significant statistical values are shown.

Finally, to approach whether the changes observed on wound closure kinetics, quality regenerated skin, and healing process from acd-MSCs of different ages could be due to the secretion levels of growth factors and specific proteins relevant to wound healing such as IGF-1, EGF, KGF, HGF, VEGF, ANG-1, ANG-2, MMP-1, and MMP-3, we analyzed and compared secretion levels from acd-MSCs of young, adult, or old age donors.  Figure 8 shows that IGF-1, PGE_2_, and HGF secretion levels did not show significant difference from acd-MSCs from donors of all three ages, whereas KGF, VEGF, ANG-I, and MPP-1 secretion levels were significantly higher only in old donors. On the other hand, CoL-1 secretion level was significantly higher in young donors. EGF, ANG-1, and MPP-3 secretion levels were not observed in acd-MSCs from different aged donors.

## 4. Discussion

Researchers have recently begun to collect data concerning the effects of natural aging on MSCs in their regenerative potential. The present work shows whether donor age modifies the regenerative potential of BM-MSCs on cutaneous wound healing model [18].

Previous studies in different animal models of regeneration such as partial hepatectomy [23], spinal cord injury [24], and critical bone defect [25] have suggested that the regenerative therapeutic effect of MSCs occurs early on in days following implant. Our findings corroborated the above, since enhanced wound closure kinetics was evident as early as two days after BM-MSCs administration from adult mouse donors, whilst this effect was less dramatic with their acd-MSCs and occurred later (day 6). These findings suggest that the effect of BM-MSCs might occur by, at least, two basic synergistically acting mechanisms and during different wound healing phases: (i) MSC transdifferentiation into epithelial cells [9] and/or (ii) the secretion of paracrine factors stimulating local dermal fibroblast and keratinocyte migration, along with a contribution towards extracellular matrix and neovascularization formation and remodeling [10]. Nevertheless, it is still controversial whether MSCs can contribute significantly to regenerate damaged tissue via tissue specific transdifferentiation, since most studies have agreed that although MSCs can migrate* in vivo* to injury sites in response to chemotactic signals, only a small percentage of engrafted MSCs actually become incorporated and survive within damaged tissue, as it was shown by Wu et al. in an excisional wound model in normal and diabetic mice [26]. Other studies have revealed that transplanted MSCs do not necessarily have to be in close proximity to damaged tissue to promote wound repair and functional recovery. It was shown by Basiouny et al. that the effect of BM-MSCs on healing of induced full thickness skin wounds in albino rats using topical and systemic injections was effective with both methods of BM-MSCs injection; however, the topical method was more effective [27].

In this context, recent and growing evidence indicates that the main MSC therapeutic action concerned in repairing skin disorders is by secretion of cytokines and growth factors that regulate local cellular response in cutaneous injury. This theory has been further reinforced by our findings and recent studies, which have shown that allogeneic and xenogeneic acd-MSCs have enhanced wound healing when administered locally into the wound from diabetic and nondiabetic mice and rats [28].

Until now, most of the studies with MSCs in cutaneous wound healing agree that these cells can accelerate wound closure due to the increase of epithelialization, granulation tissue formation, and neovascularization [29]. The foregoing facts could be due to MSCs secreting trophic factors such as EGF, KGF, IGF-1, and bFGF that are particularly related to increased dermal fibroblast migration and proliferation and stimulate keratinocyte proliferation and differentiation. In our study, a qualitative improvement, such as superior rete ridge architecture, multilayered structure, and major dermoepidermal junction, was observed in the groups treated with both doses of BM-MSCs from different ages or their acd-MSCs rather than vehicle alone.

MSCs also play an important role in healing the mesenchyme. This was confirmed by our data where increased formation of appendage-like structure (hair follicles or sebaceous glands) was observed in BM-MSCs from three ages or their acd-MSCs-treated groups. This potential benefit has been observed by other studies using BM-MSCs, human umbilical cord derived MSCs [30], or products derived of these cells such as exosomes [31] or conditioned medium [26].

Granulation tissue is a tissue of neoformation with reparative properties and it is a critical early step in healing [32]. We observed macroscopically higher granulation tissue formation on day 4 in the bed of BM-MSCs-treated wounds than wounds treated with acd-MSCs or vehicle alone (Supplementary Figure  3). In addition, our histological assessment on day 12 showed higher granulation tissue formation in the groups treated with BM-MSCs from adult age or their acd-MSCs more than BM-MSCs from young and old age or vehicle alone. The granulation tissue type in groups treated with BM-MSCs or their acd-MSCs was in more mature stage development compared to group treated with vehicle where the granulation tissue was in an earlier phase with abundant proliferation fibroblastic type. This observation was consistent with one of the MSCs' therapeutic functions regarding wounds described to date where the early induction of granulation tissue followed by neovascular network leads to fast wounds healing [32].

Greater leukocyte infiltration was found on day 12 after lesion in wounds receiving young and old BM-MSCs or their acd-MSCs or vehicle compared to adult BM-MSCs or their acd-MSCs. These results suggested that inappropriate cell source or insufficient growth factor delivery might contribute towards a chronic inflammatory state becoming established. Impaired cytokine production or immunological activity has been associated with age-related alterations [33].

Wound nonhealing is associated with atypical matrix deposition that may be related to the overproduction or insufficient growth factors [33]. It was observed in our study that wounds receiving only vehicle compared to wounds treated with BM-MSCs from three different donor ages or their acd-MSCs had more density and optimum collagenous fiber organization at the dermal matrix compared.

However, we show that BM-MSCs or their acd-MSCs contribute significantly to improve the parameters of the regenerated skin compared with wounds treated only with vehicle. Our data showed that regenerative potential of BM-MSCs or their acd-MSCs changed with donor age, where adult donors showed a higher accelerated kinetic closure and a better quality and healing regenerated skin. Nevertheless, these changes in the regenerative potential observed in the preclinical model did not have some clear relation with* in vitro* properties of BM-MSCs obtained from mouse donors of different age. Studies covering many species (rodents, monkeys, and humans) have indicated a correlation between age and declining CFU number [34]; our observations presented here also demonstrate that this effect occurs. Nevertheless, the markers used in this study to identify BM-MSCs (CD45.2, CD11b, Sca-1, CD90, and ASMA) had stable expression during MSC aging, this being consistent with other studies where the phenotype did not change with donor age [34]. Some groups have observed a significant decrease in MSC growth rate regarding old donors [35]; by contrast, others, such as Katsara et al., report that mouse BM-MSCs showed similar proliferation capacities* in vitro*, independent of age or sex, and retained their proliferative capacity for up to 5 months in culture [36]. Similar observations were made by Bergman et al. who showed that the basal proliferative rate in cultures from older animals was more than three times larger than the observed one in cultures from young animals [37]. In our study, we observed a rapid expansion of old donor BM-MSC; nevertheless, such change in proliferation potential was not statistically significant. Differences in cell morphology are often associated with senescence; some authors have reported that young donor MSCs have spindle type morphology in culture, whereas MSCs from older patients do not have them [14, 38]. Nevertheless, this change was not observed in our cultures of BM-MSCs from each age group. There is conflicting data regarding the effect of aging on MSC potential to differentiate into osteogenic, adipogenic, and other lineages; few studies have found no effect [39] but most of them suggest that aging reduces osteogenesis and chondrogenesis, whilst enhancing adipogenic potential [40]. We found that different aged donors' BM-MSCs preserved their ability regarding* in vitro* differentiation into osteogenic and adipogenic cells.

Successful wound healing is particularly related to trophic factors such as EGF, KGF, VEGF, IGF-1, bFGF, PDGF-BB, ANG-1, SDF-1, and MMP9 or cytokines such as TGF-beta, IL-6, and IL-8 [26, 41]. Recent studies have shown dramatic changes in the expression of genes in murine MSCs during aging, including those associated with growth factor and cytokines, such as VEGF, HGF, IGF-1, TGF-beta IL-6, and IL-8 [42]. Our results in the difference of the gene expression levels from BM-MSCs of the three ages were similar to those reported by Wilson et al.'s study, where genes associated with growth factors such as VEGF, HGF, G-CSF, IGF, and EGF showed markedly lower levels of transcripts in old MSCs murine and rat [42]. This difference in gene expression levels may be due to the fact that old stem cells exhibit global suppression of RNA polymerase II serine-2 phosphorylation, which triggers productive transcription elongation, mRNA processing, and release of mature mRNA [43].

Nevertheless, the difference in gene expression levels found here partly could give an explanation to the differences observed in the regenerative potential in wound healing, due to the fact that mRNA levels from adult BM-MSCs were not the lowest observed in BM-MSCs from three age groups of donors.

In order to provide evidence if the changes in the regenerative potential observed* in vivo* have some relation with paracrine profile, we compared the level of secretion of growth factors and specific proteins in acd-MSCs derived from young, adult, and old mice cultured under standard conditions. We observed significant difference in five of the ten growth factors analyzed. The acd-MSCs from old BM-MSCs had higher secretion levels of VEGF, ANG-II, KGF, HGF, and MMP-1 than the acd-MSCs from young and adult BM-MSCs. Contradictory results had been observed from other groups where MSCs young and old rats cultured had no significant difference in either cell type [44]. Moreover, in the study by Jiang et al., secretion of VEGF was significantly higher in young MSCs than in old MSCs [45]. The inconsistency with these results could be due to the source of acd-MSCs or the time window of supernatant collection.

Regarding whether different paracrine capacity of BM-MSCs from young, adult, and old donors can explain the observed differences on regenerative potential, we suggest that it is possible because it is well known that the success of the wound healing process depends on a regulated secretion of growth factors, cytokines, and chemokines involved in a complex integration of signals that coordinate cellular processes [41]. Wound nonhealing has been related to the overproduction (acute wound) or insufficient (chronic wounds) growth factors and cytokines such as EGF, FGF-2, TGF-beta, PDGF, VEGF, IL-1, IL-6, and TNF-alpha [33]. In this context we observed that the levels of secreted growth factors and specific proteins from adult BM-MSCs were not as low as they were from young BM-MSCs or as high as from old BM-MSCs. This observation could be indicative of a regulation of secretion of the growth factors and specific proteins analyzed, which are key in the wound healing process.

## 5. Conclusions

Our data show that MSC efficacy was negatively affected by donor age and that this fact might have been because of changes in their paracrine factor expression. So, increased BM-MSCs age should therefore be considered as a risk regarding the effectiveness of any therapeutic application of BM-MSCs or their acd-MSCs, especially concerning autologous cell therapy for elderly patients.

## Supplementary Material

Supplementary Figure 1: We standardized histological criteria in haematoxylin-eosin to provide quantitative analysis regarding the dermoepidermal junction and degree of leukocyte infiltration.
Supplementary Figure 2: We evaluate reepithelialization with an epithelial tissue marker (pan-cytokeratin). We found expression in all groups.
Supplementary Figure 3: We evaluate granulation tissue that is a tissue of neoformation with reparative properties. We observed macroscopically higher granulation tissue formation on day 4 in the bed of BM-MSCs-treated wounds than wounds treated with acd-MSCs or vehicle alone.
Supplementary Table 1: Statistical data of flow cytometry showed that no significant age-related changes in the marker expression levels between the three age groups.
Supplementary Table 2: List with the specific primer.
