# Supplementary material for: Regenerative Potential of Mesenchymal Stromal Cells: Age-Related Changes
Source: Stem Cells Int. 2016 May 9;2016:1461648. doi: 10.1155/2016/1461648 (PMC4876257; doi:10.1155/2016/1461648)
Supplement: Supplementary file 1 — Supplementary Figure 1: We standardized histological criteria in haematoxylin-eosin to provide quantitative analysis regarding the dermoepidermal junction and degree of leukocyte infiltration. Supplementary Figure 2: We evaluate reepithelialization with an epithelial tissue marker (pan-cytokeratin). We found expression in all groups. Supplementary Figure 3: We evaluate granulation tissue that is a tissue of neoformation with reparative properties. We observed macroscopically higher granulation tissue formation on day 4 in the bed of BM-MSCs-treated wounds than wounds treated with acd-MSCs or vehicle alone. Supplementary Table 1: Statistical data of flow cytometry showed that no significant age-related changes in the marker expression levels between the three age groups. Supplementary Table 2: List with the specific primer. [file 1461648.f1.pdf]

Supporting Information Legends

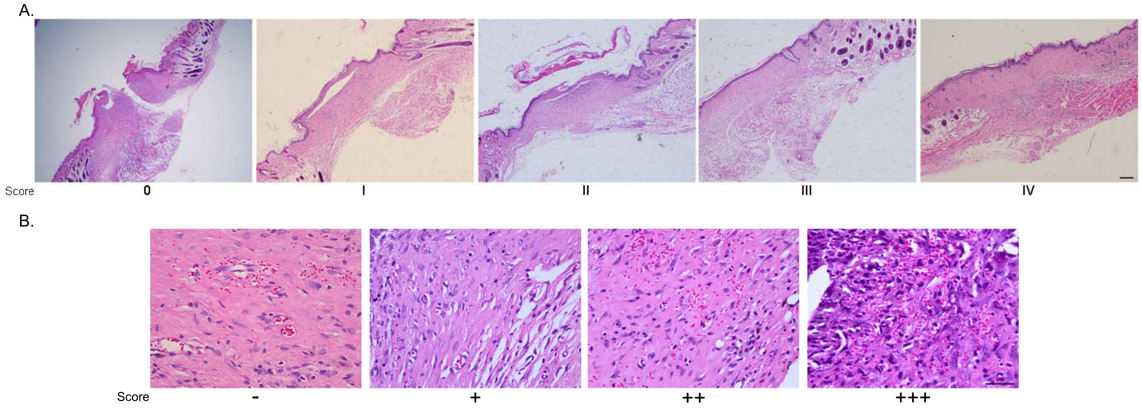

Supplementary figure 1. (A) Dermo-epidermal junction histological scores. Haematoxylin-eosin stained that represents the dermo-epidermal junction. Image J analysis software was used for estimating unbound area size.

0: > 2,801 area per pixels represents an incomplete junction

I: 1,201-2,800 area per pixels

II: 751-1,200 area per pixels

III: 401-750 area per pixels

IV: 0.1-400 area per pixels represents a complete junction

Scale bar 100 µm.

(B) Histological scores regarding leukocyte infiltration level. (-) absent, (+) mild, (++) moderate, (+++) severe. Scale bar 50 µm

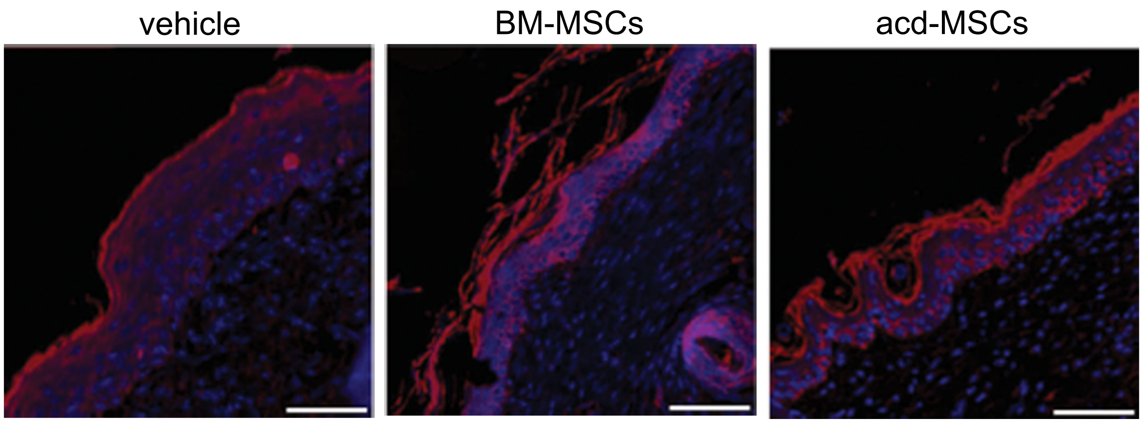

Supplementary figure 2. Pan-cytokeratin expression 12 days after treatment. Keratinocytes were stained with a monoclonal antibody against epidermal keratin subunits and visualized with a secondary antibody: Alexa Fluor 647. DAPI was used for nuclear staining. The representative results of 5-15 animals per experimental group. Scale bar 50 µm.

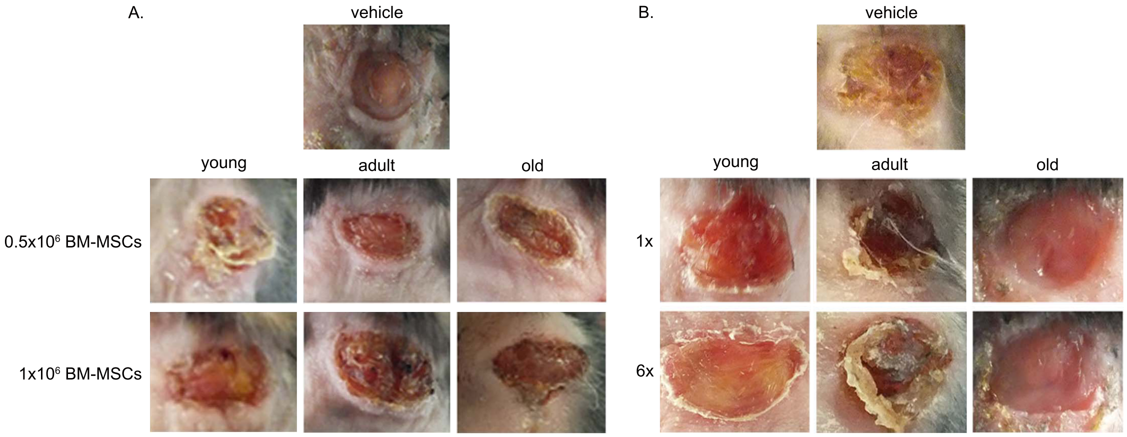

Supplementary figure 3. Granulation tissue formation (A) After 4 days the bed of a wound treated with bmMSCs isolated from young, adult and old female C57BL/6 donors and **(B)** the bed of a wound treated with acd-MSCs.

Tables

Supplementary table 1. Statistical data of flow cytometry.

Percentage of cells positive for the marker

Age	CD90.2	Sca-1	ASMA
**Young**	65.2	75.7	95.4
**Adult**	63.8	77.3	96.1
**Old**	60.2	93.7	88.3

Mean fluorescence intensity (isotype / marker)

Age	CD90.2		Sca-1		ASMA		CD45.2		CD11b	
**Young**	21.5	73.6	7.8	126.5	6.8	387.5	5.3	10.9	5.1	7.8
**Adult**	16.1	49.4	4.2	59.2	6.8	922.0	4.2	4.6	5.1	6.8
**Old**	16.8	46.0	2.8	162.8	5.3	57.1	4.2	5.9	7.0	11.3

Supplementary table 2. Primer and amplicon characteristics.

Gene	GenBank	Sequences 5′→3′	Tm (°C)	Amplicon size (bp)	Reference
KGF (FGF-7)	NM_008008.4	F-TGAGTCCGGAGCAAACGGCT<br>R-TGAGTCCGGAGCAAACGGCT	86	131	[1]
IGF-1	NM_010512.3	F-TGGATGCTCTTCAGTTCGTG<br>R-GCTTTGGGCATGTCAGTGTG	88	220	[2]
HGF	NM_001289458.1	F-TCTGCTCGAACTGCAAGCATGA<br>R-TCCTGATACACCTGTTGGCACACT	86	250	http://pga.mgh.harvard.edu/primerbank/ PRIMER BANK
VEGF alpha	NM_001025257.3	F-AGGAGAGCAGAAGTCCCATGAAGT<br>R-GGTGAGGTTTGATCCGCATGATCT	88	242	[3]
VEGF delta	NM_010216.1	F-CCGGTTGAAGCTCAAAAGTCTTGC<br>R-CCTCCACACCGGAAGACATTTACA	86	231	[3]
ANG 1	XM_006520323.1	F-TGCACTAAAGAAGGTGTTTTGCT<br>R-CCTCCCCCATTCACATCCATATT	83	176	http://pga.mgh.harvard.edu/primerbank/ PRIMER BANK
ANG 2	NM_007426.4	F-CCTCGACTACGACGACTCAGT<br>R-TCTGCACCACATTCTGTTGGA	84	148	http://pga.mgh.harvard.edu/primerbank/ PRIMER BANK
Collagen type1	NM_007742.3	F-AGAACATCACCTATCACTGCAAGA<br>R-GTGGTTTTGTATTCGATGACTGTCT	89º	205	[4]
MMP1	NM_008607.2	F-GAACATCCATCCCGTGACCTT<br>R-AAGAGGGTCTTCCCCGTCTT	85	173	our design PRIMER PREMIER 5.0
MMP3	NM_010809.1	F-ACATGGAGACTTTGTCCCTTTTG<br>R-TTGGCTGAGTGGTAGAGTCCC	86	192	http://pga.mgh.harvard.edu/primerbank/ PRIMER BANK
GAPDH	NM_001289726.1	F-ACTCCACTCACGGCAAATTC<br>R-TCTCCATGGTGGTGAAGACA	86	171	[5]
